# Immune responses upon *Campylobacter jejuni* infection of secondary abiotic mice lacking nucleotide-oligomerization-domain-2

**DOI:** 10.1186/s13099-017-0182-0

**Published:** 2017-06-06

**Authors:** Stefan Bereswill, Ursula Grundmann, Marie E. Alutis, André Fischer, Anja A. Kühl, Markus M. Heimesaat

**Affiliations:** 1 Department of Microbiology and Hygiene, Charité-University Medicine Berlin, CC5, Campus Benjamin Franklin, FEM, Garystr. 5, 14195 Berlin, Germany; 2 Research Center ImmunoSciences (RCIS), Charité-University Medicine Berlin, Berlin, Germany

**Keywords:** *Campylobacter jejuni*, Nucleotide-oligomerization-domain-2 (Nod2), Secondary abiotic (gnotobiotic) mice, Bacterial colonization properties, Pro-inflammatory cytokines, Adaptive immune cells, IL-23/IL-22/IL-18 axis, Apoptosis, Bacterial translocation, Mucin-2

## Abstract

**Background:**

*Campylobacter jejuni* infections are of rising importance worldwide. Given that innate immune receptors including nucleotide-oligomerization-domain-2 (Nod2) are essentially involved in combating enteropathogenic infections, we here surveyed the impact of Nod2 in murine campylobacteriosis.

**Methods and results:**

In order to overcome physiological colonization resistance preventing from *C. jejuni* infection, we generated secondary abiotic Nod2^−/−^ and wildtype (WT) mice by broad-spectrum antibiotic treatment. Mice were then perorally infected with *C. jejuni* strain 81-176 on 2 consecutive days and could be stably colonized by the pathogen at high loads. Notably, Nod2 deficiency did not affect gastrointestinal colonization properties of *C. jejuni*. Despite high intestinal pathogenic burdens mice were virtually uncompromised and exhibited fecal blood in single cases only. At day 7 postinfection (p.i.) similar increases in numbers of colonic epithelial apoptotic cells could be observed in mice of either genotype, whereas *C. jejuni* infected Nod2^−/−^ mice displayed more distinct regenerative properties in the colon than WT controls. *C. jejuni* infection was accompanied by increases in distinct immune cell populations such as T lymphocytes and regulatory T cells in mice of either genotype. Increases in T lymphocytes, however, were less pronounced in large intestines of Nod2^−/−^ mice at day 7 p.i. when compared to WT mice, whereas colonic numbers of B lymphocytes were elevated in WT controls only upon *C. jejuni* infection. At day 7 p.i., colonic pro-inflammatory mediators including nitric oxide, TNF, IFN-γ and IL-22 increased more distinctly in Nod2^−/−^ as compared to WT mice, whereas *C. jejuni* induced IL-23p19 and IL-18 levels were lower in the large intestines of the former. Converse to the colon, however, ileal concentrations of nitric oxide, TNF, IFN-γ, IL-6 and IL-10 were lower in Nod2^−/−^ as compared to WT mice at day 7 p.i. Even though MUC2 was down-regulated in *C. jejuni* infected Nod2^−/−^ mice, this did not result in increased pathogenic translocation from the intestinal tract to extra-intestinal compartments.

**Conclusion:**

In secondary abiotic mice, Nod2 signaling is involved in the orchestrated host immune responses upon *C. jejuni* infection, but does not control pathogen loads in the gastrointestinal tract.

**Electronic supplementary material:**

The online version of this article (doi:10.1186/s13099-017-0182-0) contains supplementary material, which is available to authorized users.

## Background

Human *Campylobacter jejuni* infections have been progressively rising during the past decades [1–3]. A plethora of wild and domestic animals harbor *C. jejuni* as commensal bacteria within their intestinal microbiota. Upon zoonotic transmission from livestock animals humans become infected via consumption of contaminated meat or surface water [4–6]. Infected individuals may be asymptomatic, present with rather mild malaise and watery diarrhea or with full blown disease characterized by acute enterocolitis with abdominal cramps, fever, and bloody, inflammatory diarrhea lasting for days or even weeks [1]. Whereas disease is self-limiting in the vast majority of cases, post-infectious sequelae affecting the joints (i.e. reactive arthritis), the nervous system (i.e. Guillain–Barré syndrome, Miller Fisher syndrome and Bickerstaff encephalitis) or the intestinal tract (i.e. irritable bowel syndrome) might arise sporadically [1, 3, 7]. Susceptibility to pathogenic infection is highly depending on the distinct intestinal microbiota composition of the respective host. Conventionally colonized adult mice, for instance, are protected from *C. jejuni* infection and expel the pathogen within a few days even upon peroral high dose infection [8–10]. Modification of the murine microbiota by broad-spectrum antibiotic treatment, however, compromizes physiological colonization resistance and facilitates stable gastrointestinal *C. jejuni* infection of the resulting secondary abiotic mice at high loads [8, 10, 11]. In turn, secondary abiotic mice present with distinct *C. jejuni* induced pro-inflammatory immune and colonic apoptotic responses thereby mimicking key features of human campylobacteriosis [6, 8]. Hence, the secondary abiotic mouse model has been proven well-suitable to dissect enteropathogenic-host interactions in vivo [10, 11].

Host immune responses are pivotal for combating enteropathogenic including *C. jejuni* infections. The nucleotide-binding oligomerization domain (Nod)-like receptors comprize an important family of signaling molecules sensing microbial products and damage-associated factors and thus contributing to innate immunity [12]. Among these, Nod2 is expressed by innate (such as macrophages and dendritic cells) and adaptive immune cell subsets including T lymphocytes as well as by Paneth cells [13–15]. Upon activation by muramyl dipeptide (MDP), a constituent of bacterial peptidoglycans, Nod2 confers resistance to a broad variety of bacterial species [12, 16–18]. To date it is still unclear, however, whether Nod2 can also sense other microbial molecules or whether it rather acts as a mere signaling partner [19]. The importance of Nod2 in sensing and elimination of enteropathogens have been demonstrated in vivo, given that Nod2^−/−^ mice were more susceptible to *Listeria monocytogenes* or *Yersinia pseudotuberculosis* infection [20, 21].

In the present study we investigated the impact of Nod2 in *C. jejuni* infection of secondary abiotic mice. We addressed whether pathogenic colonization properties, potential translocation of viable bacteria to extra-intestinal compartments, infection induced macroscopic and microscopic sequelae, and pro-inflammatory intestinal as well as systemic immune responses were affected by Nod2 signaling in *C. jejuni* infected secondary abiotic mice.

## Methods

### Ethics statement

All animal experiments were conducted according to the European Guidelines for animal welfare (2010/63/EU) with approval of the commission for animal experiments headed by the “Landesamt für Gesundheit und Soziales” (LaGeSo, Berlin, registration number G0135/10). Animal welfare was monitored twice daily by assessment of clinical conditions.

### Generation of secondary abiotic mice and *C. jejuni* infection

Female Nod2 deficient (Nod2^−/−^) mice (in C57BL/6j background) and matched wildtype mice were reared and maintained within the same specific pathogen free (SPF) unit of the Forschungseinrichtungen für Experimentelle Medizin (FEM, Charité-University Medicine Berlin). In order to overcome physiological colonization resistance and thus assure stable pathogenic colonization of the gastrointestinal tract [8], secondary abiotic (i.e. gnotobiotic) mice virtually lacking an intestinal microbiota were generated upon broad-spectrum antibiotic treatment for at least 8 weeks as described earlier [8, 22]. Three days before infection, the antibiotic cocktail was replaced by sterile water. Mice were then perorally infected with 10^9^ colony forming units (CFU) of viable *C. jejuni* strain 81-176 in a volume of 0.3 mL phosphate buffered saline (PBS; Gibco, life technologies, UK) on two consecutive days (days 0 and 1) by gavage as described previously [8]. To prevent mice from contaminations, animals were continuously kept in a sterile environment (autoclaved food and drinking water or sterile antibiotic cocktail) and handled under strict aseptic conditions.

### Clinical conditions

To assess clinical signs of *C. jejuni* induced infection a standardized cumulative clinical score (maximum 12 points), addressing the occurrence of blood in feces (0: no blood; 2: microscopic detection of blood by the Guajac method using Haemoccult, Beckman Coulter/PCD, Germany; 4: macroscopic blood visible), diarrhea (0: formed feces; 2: pasty feces; 4: liquid feces), and the clinical aspect (0: normal; 2: ruffled fur, less locomotion; 4: isolation, severely compromized locomotion, pre-final aspect) was used on a daily basis as described earlier [23–25].

### Sampling procedures and histopathology

Mice were sacrificed at day 7 postinfection (p.i.) by isoflurane treatment (Abbott, Greifswald, Germany). Intestinal ex vivo biopsies were asserved under sterile conditions and collected from each mouse in parallel for microbiological, immunohistopathological and immunological analyses. Histopathological changes were determined in samples derived from the colon that were immediately fixed in 5% formalin and embedded in paraffin. Sections (5 μm) were stained with hematoxylin and eosin (H&E), examined by light microscopy (magnification ×100 and ×400) and histopathological changes quantitatively assessed applying a standardized histopathological scoring system (maximum 4 points) as described previously [26].

### Immunohistochemistry

In situ immunohistochemical analysis of colonic paraffin sections was performed as stated elsewhere [24, 25, 27, 28]. Primary antibodies against cleaved caspase-3 (Asp175, Cell Signaling, USA, 1:200), Ki67 (TEC3; Dako, Denmark; 1:100), CD3 (#N1580; Dako; 1:10), FOXP3 (FJK-16s; eBioscience, Germany; 1:100) and B220 (eBioscience; 1:200) were used. For each animal, the average number of positively stained cells within at least six high power fields (HPF, 0.287 mm^2^, ×400 magnification) were determined lightmicroscopically by a blinded investigator.

### Quantitative analysis of bacterial colonization

Viable *C. jejuni* were quantitatively assessed in feces over time p.i. or at time of necropsy (i.e. day 7 p.i.) in luminal samples taken from the gastrointestinal tract (i.e. stomach, duodenum, ileum and colon) and in homogenates of ex vivo biosies taken from mesenteric lymph nodes (MLN), spleen, liver and kidney, dissolved in sterile PBS and serial dilutions streaked onto Karmali- and Columbia-Agar supplemented with 5% sheep blood (Oxoid) for two days at 37 °C under microaerobic conditions using CampyGen gas packs (Oxoid). The respective weights of fecal or tissue samples were determined by the difference of the sample weights before and after asservation. The detection limit of viable pathogens was ≈100 CFU per g.

### Cytokine detection in supernatants of intestinal and extra-intestinal ex vivo biopsies

Colonic and ileal ex vivo biopsies were cut longitudinally and washed in PBS. MLN, spleen or strips of approximately 1 cm^2^ respective intestinal tissues were placed in 24-flat-bottom well culture plates (Nunc, Wiesbaden, Germany) containing 500 μL serum-free RPMI 1640 medium (Gibco, life technologies, Paisley, UK) supplemented with penicillin (100 U/mL) and streptomycin (100 µg/mL; PAA Laboratories). After 18 h at 37 °C, culture supernatants were tested for TNF, IFN-γ, MCP-1, IL-12p70, IL-6 and IL-10 by the Mouse Inflammation Cytometric Bead Assay (CBA; BD Biosciences, Heidelberg, Germany) on a BD FACSCanto II flow cytometer (BD Biosciences). Nitric oxide (NO) was measured by Griess reaction as described earlier [22].

### Real-time PCR

RNA was isolated from snap frozen colonic ex vivo biopsies, reverse transcribed and analyzed as described previously [29]. Murine mucin-2 (MUC2), IL-23p19, IL-22, and IL-18 mRNA expression was analyzed using Light Cycler Data Analysis Software (Roche). The mRNA of the housekeeping gene for hypoxanthine-phosphoribosyltransferase (HPRT) was used as reference, and the mRNA expression levels of the individual genes were normalized to the lowest measured value and expressed as fold expression (Arbitrary Units).

### Statistical analysis

Medians, means and levels of significance were determined using Mann–Whitney test (GraphPad Prism v5, La Jolla, CA, USA) as indicated. Two-sided probability (p) values ≤0.05 were considered significant.

## Results

### *Campylobacter jejuni* colonization alongside the gastrointestinal tract and clinical conditions in secondary abiotic Nod2^−/−^ mice

**Fig. 1:**
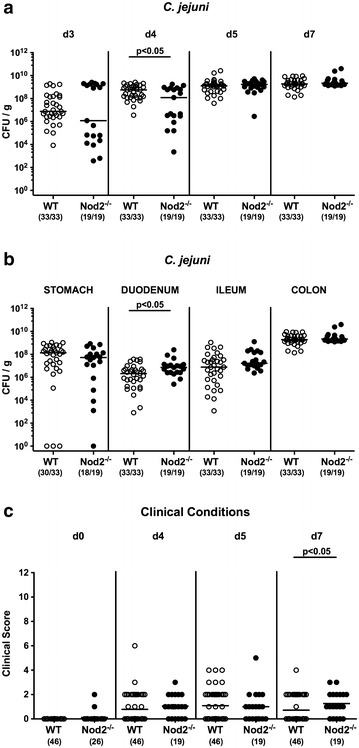
Gastrointestinal colonization densities and clinical conditions in *C. jejuni* infected secondary abiotic mice Nod2^−/−^ mice. Secondary wildtype (WT; *white circles*) and Nod2^−/−^ mice (*black circles*) were generated by broad-spectrum antibiotic treatment and perorally infected with *C. jejuni* strain 81-176 by gavage at day (d) 0 and d1. Pathogenic loads (CFU, colony forming units per gram) were assessed in **a** fecal samples over time postinfection as indicated and in **b** defined parts of the gastrointestinal tract at d7 p.i. by culture. Numbers of mice harboring *C. jejuni* out of the total number of analyzed animals are given in *parentheses*. **c** Clinical symptoms were assessed before and after infection applying a standardized clinical scoring system (see “Methods”). Medians (*black bars*), numbers of mice (in *parentheses*) and levels of significance (p-values) determined by Mann–Whitney U test are indicated. Data were pooled from at least three independent experiments

Given that the intestinal microbiota prevents the murine host from stable *C. jejuni* infection [8, 10], we generated secondary abiotic Nod2^−/−^ mice and WT counterparts by broad-spectrum antibiotic treatment in order to overcome physiological colonization resistance. Following peroral infection with 10^9^ viable bacteria of *C. jejuni* strain 81-176 on 2 consecutive days (i.e. days 0 and 1) by gavage, the pathogen could stably establish within the gastrointestinal tract with highest loads of approximately 10^9^ CFU per g in the colon at day 7 p.i. (Fig. 1a, b). Nod2 did not impact colonization properties in a biologically relevant fashion as indicated by similar intestinal *C. jejuni* loads in infected Nod2^−/−^ and WT mice (Fig. 1a, b). Fecal *C. jejuni* counts were slightly lower (approximately one order of magnitude) in Nod2^−/−^ as compared to WT mice at day 4 p.i. (p < 0.05; Fig. 1a), whereas at necropsy, duodenal pathogenic burdens were even marginally higher (approximately 0.5 log) in the former (p < 0.05; Fig. 1b).

We further surveyed whether Nod2 deficiency had an impact on clinical conditions upon murine *C. jejuni* infection. To address this, we applied a cumulative clinical score assessing gross clinical aspects of mice, fecal consistency and abundance of blood in stool samples. Overall, mice of either genotype were virtually uncompromised upon *C. jejuni* infection (Fig. 1c). As early as 5 days p.i., 23.9 and 10.5% of WT and Nod2^−/−^ mice, respectively, displayed bloody stool microscopically or even macroscopically, whereas respective fecal blood positivity rates were 8.7 and 10.5% at day 7 p.i. (Additional file 1: Figure S1).

### Microscopic inflammatory changes in large intestines of *C. jejuni* infected secondary abiotic Nod2^−/−^ mice

**Fig. 2:**
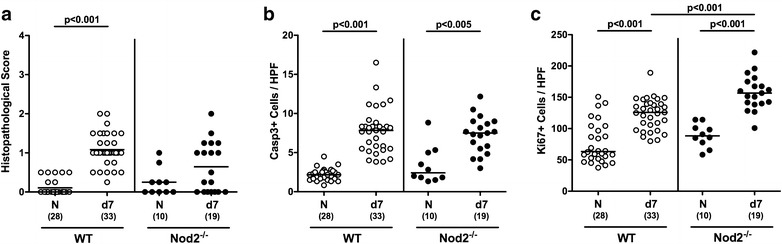
Microscopic large intestinal changes in *C. jejuni* infected secondary abiotic Nod2^−/−^ mice. Secondary abiotic wildtype (WT; *white circles*) and Nod2^−/−^ mice (*black circles*) were generated by broad-spectrum antibiotic treatment and perorally infected with *C. jejuni* strain 81-176 by gavage at day (d) 0 and d1. **a** Histopathological mucosal changes were assessed in H&E stained large intestinal paraffin sections. Furthermore, the average number of colonic epithelial **b** apoptotic cells (positive for caspase-3, Casp3) and **c** proliferating cells (positive for Ki67) from six high power fields (HPF, ×400 magnification) per animal was determined microscopically in immunohistochemically stained colonic paraffin sections at day 7 following *C. jejuni* infection. Naive (N) secondary abiotic mice served as uninfected controls. Medians (*black bars*), levels of significance (p-values) determined by Mann–Whitney U test and numbers of analyzed animals (in *parentheses*) are indicated. Data were pooled from four independent experiments

We next determined potential Nod2 dependent histopathological sequelae of *C. jejuni* infection in colonic paraffin sections applying a standardized histopathological scoring system. Slightly higher histopathological scores could be assessed in the large intestines of *C. jejuni* infected as compared to naive WT mice indicative for single isolated to mild scattered cell infiltrates within the mucosa (p < 0.001; Fig. 2a; Additional file 2: Figure S2). In Nod2^−/−^ mice a trend towards *C. jejuni* induced increases in colonic histopathological scores could be observed at day 7 p.i. (n.s. versus naive; Fig. 2a; Additional file 2: Figure S2). Since apoptosis constitutes an established marker for the microscopic evaluation of intestinal inflammation in murine campylobacteriosis [8], we stained colonic paraffin sections with caspase-3 antibodies. As compared to naive counterparts, colonic epithelial apoptotic cell numbers increased more than threefold until day 7 following *C. jejuni* infection of Nod2^−/−^ and WT mice (p < 0.005 and p < 0.001, respectively; Fig. 2b; Additional file 3: Figure S3). Increases in apoptotic cell numbers were accompanied by elevated colonic epithelial Ki67 positive cell counts at day 7 p.i. (p < 0.001 vs naive; Fig. 2c; Additional file 4: Figure S4), indicative for proliferative/regenerative measures counteracting potential cell damage. Notably, Ki67 positive cell numbers were higher in infected Nod2^−/−^ as compared to WT mice (p < 0.001; Fig. 2c; Additional file 4: Figure S4). Hence, comparable clinical sequelae of *C. jejuni* infection of Nod2^−/−^ and WT mice were paralleled by similar microscopic inflammatory changes within the infected large intestines, whereas *C. jejuni* infected Nod2^−/−^ mice exhibited more distinct regenerative properties in the colon than WT controls.

### Colonic immune cell responses in *C. jejuni* infected secondary abiotic Nod2^−/−^ mice

**Fig. 3:**
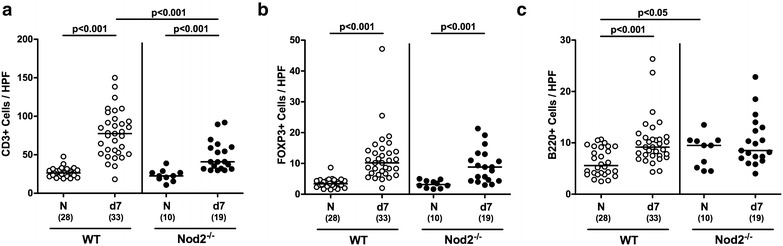
Large intestinal immune cell populations in *C. jejuni* infected secondary abiotic Nod2^−/−^ mice. Secondary abiotic wildtype (WT; *white circles*) and Nod2^−/−^ mice (*black circles*) were generated by broad-spectrum antibiotic treatment and perorally infected with *C. jejuni* strain 81-176 by gavage at day (d) 0 and d1. The average number of colonic epithelial **a** T lymphocytes (positive for CD3), **b** regulatory T cells (Treg; positive for FOXP3) and **c** B lymphocytes (positive for B220) from six high power fields (HPF, ×400 magnification) per animal was determined microscopically in immunohistochemically stained colonic paraffin sections at day 7 following *C. jejuni* infection. Naive (N) secondary abiotic mice served as uninfected controls. Medians (*black bars*), levels of significance (p-values) determined by Mann–Whitney U test and numbers of analyzed animals (in *parentheses*) are indicated. Data were pooled from four independent experiments

Given that recruitment of immune cells to the site of infection is a hallmark of intestinal inflammation in murine *C. jejuni* infection models [8], we next quantitatively assessed distinct immune cell populations in colonic paraffin sections applying in situ immunohistochemistry. Until day 7 p.i., colonic T lymphocytes increased more than threefold in WT control animals, but less distinctly in Nod2^−/−^ mice (p < 0.001; Fig. 3a; Additional file 5: Figure S5). Whereas mice of either genotype displayed elevated Treg numbers in their large intestines at day 7 p.i. (p < 0.001 vs naive; Fig. 3b; Additional file 6: Figure S6), B lymphocytes increased in *C. jejuni* infected WT mice only (p < 0.001; Fig. 3c; Additional file 7: Figure S7). Interestingly, naive secondary abiotic Nod2^−/−^ mice exhibited higher B cell counts as compared to WT counterparts (p < 0.05; Fig. 3c; Additional file 7: Figure S7) that did not further increase upon *C. jejuni* infection of the former (n.s.; Fig. 3c). Hence, Nod2^−/−^ mice displayed less pronounced *C. jejuni* induced increases in T lymphocytes.

### Colonic cytokine secretion in *C. jejuni* infected secondary abiotic mice lacking Nod2

**Fig. 4:**
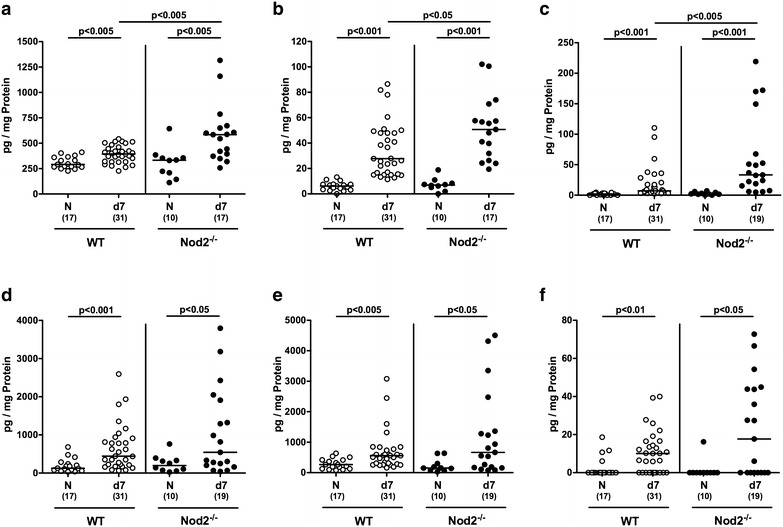
Colonic secretion of pro- and anti-inflammatory cytokines in *C. jejuni* infected secondary abiotic Nod2^−/−^ mice. Secondary abiotic wildtype (WT; *white circles*) and Nod2^−/−^ mice (*black circles*) were generated by broad-spectrum antibiotic treatment and perorally infected with *C. jejuni* strain 81-176 by gavage at day (d) 0 and d1. **a** Nitric oxide, **b** TNF, **c** IFN-γ, **d** MCP-1, **e** IL-6 and **f** IL-10 concentrations were determined in supernatants of colonic ex vivo biopsies at day 7 postinfection. Naive (N) secondary abiotic mice served as uninfected controls. Medians (*black bars*), level of significance (p-value) determined by Mann–Whitney U test and numbers of analyzed animals (in *parentheses*) are indicated. Data were pooled from four independent experiments

We next surveyed *C. jejuni* induced pro-inflammatory cytokine secretion in colonic ex vivo biopsies. At day 7 p.i., pro-inflammatory mediators such as nitric oxide, TNF, IFN-γ, MCP-1 and IL-6 were elevated in large intestines of both Nod2^−/−^ and WT mice (p < 0.05–0.001; Fig. 4a–e). *C. jejuni* induced increases in nitric oxide, TNF and IFN-γ were, however, more pronounced in Nod2^−/−^ mice as compared to WT counterparts (p < 0.05–0.005; Fig. 4a–c). Notably, *C. jejuni* infection resulted not only in enhanced pro-inflammatory, but also increased anti-inflammatory cytokine responses as indicated by elevated IL-10 levels in colonic ex vivo biopsies derived from both Nod2^−/−^ and WT mice at day 7 p.i. (p < 0.05 and p < 0.01, respectively; Fig. 4f). Hence, *C. jejuni* induced increases in large intestinal pro-inflammatory cytokines were higher in Nod2^−/−^ as compared to WT mice.

### Colonic cytokines of the IL-23/IL-22/IL-18 axis in *C. jejuni* infected secondary abiotic Nod2^−/−^ mice

**Fig. 5:**
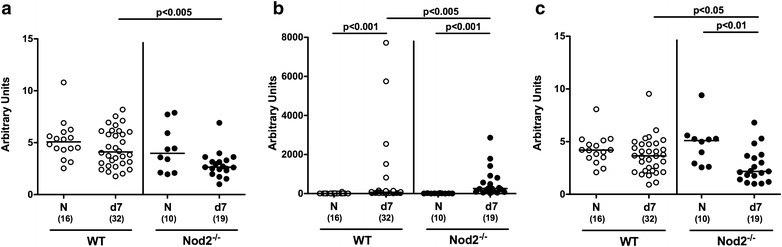
Colonic expression of cytokines belonging to the IL-23/IL-22/IL-18 axis in *C. jejuni* infected secondary abiotic Nod2^−/−^ mice. Secondary abiotic wildtype (WT; *white circles*) and Nod2^−/−^ mice (*black circles*) were generated by broad-spectrum antibiotic treatment and perorally infected with *C. jejuni* strain 81-176 by gavage at day (d) 0 and d1. **a** IL-23p19, **b** IL-22 and **c** IL-18 mRNA levels were determined in colonic ex vivo biopsies at day 7 postinfection by Real Time PCR and expressed in Arbitrary Units (fold expression). Naive (N) secondary abiotic mice served as uninfected controls. Medians (*black bars*), level of significance (p-value) determined by Mann–Whitney U test and numbers of analyzed animals (in *parentheses*) are indicated. Data were pooled from four independent experiments

Recently our group showed that cytokines belonging to the IL-23/IL-22/IL-18 axis are involved in mediating murine *C. jejuni* infection [25, 30–32]. We therefore determined expression levels of respective cytokines upon *C. jejuni* infection of secondary abiotic Nod2^−/−^ mice. Whereas colonic IL-23p19 mRNA levels were comparable in naive and *C. jejuni* infected mice of either genotype (n.s.; Fig. 5a), IL-22 was up-regulated in the large intestines of both Nod2^−/−^ and WT mice at day 7 p.i. (p < 0.001; Fig. 5b). Moreover, *C. jejuni* infection resulted in down-regulation of colonic IL-18 mRNA in Nod2^−/−^ mice only (p < 0.01; Fig. 5c). Whereas colonic IL-23p19 and IL-18 mRNA levels were lower in *C. jejuni* infected Nod2^−/−^ mice as compared to WT counterparts (p < 0.005 and p < 0.05, respectively; Fig. 5a, c), IL-22 expression was higher in the large intestines of Nod2^−/−^ versus WT mice at day 7 p.i. (p < 0.005; Fig. 5b). Hence, cytokines of the IL-23/IL-22/IL-18 axis are differentially expressed upon *C. jejuni* infection of secondary abiotic Nod2^−/−^ mice.

### Ileal cytokine secretion in *C. jejuni* infected secondary abiotic Nod2^−/−^ mice

**Fig. 6:**
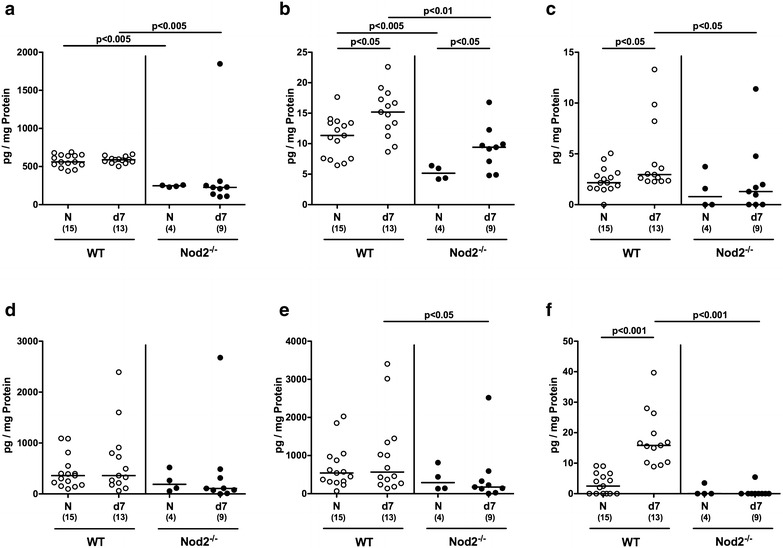
Ileal secretion of pro- and anti-inflammatory cytokines in *C. jejuni* infected secondary abiotic Nod2^−/−^ mice. Secondary abiotic wildtype (WT; *white circles*) and Nod2^−/−^ mice (*black circles*) were generated by broad-spectrum antibiotic treatment and perorally infected with *C. jejuni* strain 81-176 by gavage at day (d) 0 and d1. **a** Nitric oxide, **b** TNF, **c** IFN-γ, **d** MCP-1, **e** IL-6 and **f** IL-10 concentrations were determined in supernatants of ileal ex vivo biopsies at day 7 postinfection. Naive (N) secondary abiotic mice served as uninfected controls. Medians (*black bars*), level of significance (p-value) determined by Mann–Whitney U test and numbers of analyzed animals (in *parentheses*) are indicated. Data were pooled from four independent experiments

We next addressed whether *C. jejuni* induced cytokine secretion could also be observed in the small intestinal tract. In fact, ileal TNF concentrations were higher in mice of either genotype at day 7 p.i. as compared to respective naive counterparts (p < 0.05; Fig. 6b), whereas TNF and nitric oxide levels were lower in both naive and *C. jejuni* infected Nod2^−/−^ as compared to WT controls (p < 0.01–0.005; Fig. 6a, b). Furthermore, IFN-γ and IL-10 concentrations increased until 7 p.i. of WT, but not Nod2^−/−^ mice and were lower in the latter (p < 0.05 and p < 0.001, respectively; Fig. 6c, f). In addition, IL-6 levels were lower in the ilea of Nod2^−/−^ as compared to WT mice at day 7 p.i. (p < 0.05; Fig. 6e). Hence, converse to the colon, ileal secretion of distinct cytokines was less pronounced in *C. jejuni* infected Nod2^−/−^ mice versus WT controls.

### Pathogenic translocation in *C. jejuni* infected secondary abiotic Nod2^−/−^ mice

**Fig. 7:**
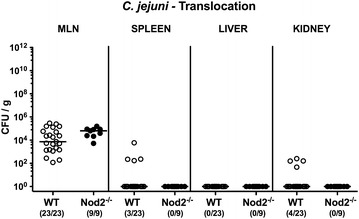
Pathogenic translocation in *C. jejuni* infected secondary abiotic Nod2^−/−^ mice. Secondary abiotic wildtype (WT; *white circles*) and Nod2^−/−^ mice (*black circles*) were generated by broad-spectrum antibiotic treatment and perorally infected with *C. jejuni* strain 81-176 by gavage at day (d) 0 and d1. Translocating pathogens were assessed in ex vivo biopsies derived from mesenteric lymph nodes (MLN), spleen, liver and kidney at day 7 postinfection by culture. Medians (*black bars*) and numbers of mice harboring *C. jejuni* out of the total number of analyzed animals (in *parentheses*) are indicated. Data were pooled from four independent experiments

We next addressed whether viable *C. jejuni* could also be isolated from extra-intestinal compartments. Whereas *C. jejuni* was detectable in MLN of all Nod2^−/−^ and WT mice at day 7 p.i., the pathogen translocated to spleen and kidney of WT mice in 13.0 and 17.4% of cases, respectively, but could not be cultured from respective organs of Nod2^−/−^ mice by direct plating (Fig. 7). Notably, irrespective of the genotype of mice, livers were free of viable *C. jejuni* (Fig. 7).

**Fig. 8:**
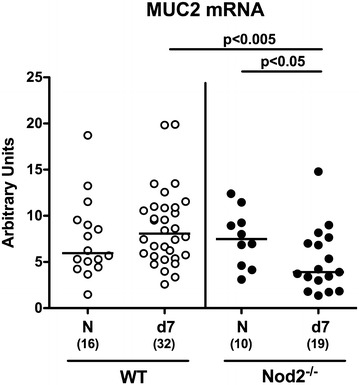
Colonic mucin-2 expression in *C. jejuni* infected secondary abiotic Nod2^−/−^ mice. Secondary abiotic wildtype (WT; *white circles*) and Nod2^−/−^ mice (*black circles*) were generated by broad-spectrum antibiotic treatment and perorally infected with *C. jejuni* strain 81-176 by gavage at day (d) 0 and d1. Mucin-2 (MUC2) mRNA levels were determined in colonic ex vivo biopsies at day 7 postinfection by Real Time PCR and expressed in Arbitrary Units (fold expression). Naive (N) secondary abiotic mice served as uninfected controls. Medians (*black bars*), level of significance (p-value) determined by Mann–Whitney U test and numbers of analyzed animals (in *parentheses*) are indicated. Data were pooled from four independent experiments

Given that mucins constitute a pivotal part of the mucus layer warranting epithelial barrier integrity and hence preventing from bacterial translocation [33, 34], we next measured mucin-2 (MUC2) expression levels in colonic ex vivo biopsies derived from *C. jejuni* infected secondary abiotic Nod2^−/−^ mice. MUC2 expression was down-regulated in the colon upon *C. jejuni* infection of Nod2^−/−^, but not WT mice (p < 0.05; Fig. 8), and colonic MUC2 mRNA levels were lower in the former versus the latter at day 7 p.i. (p < 0.005; Fig. 8). Hence, even though MUC2 was down-regulated in *C. jejuni* infected Nod2^−/−^ mice, this did not result in increased pathogenic translocation from the intestinal tract to extra-intestinal compartments.

### Cytokine responses in mesenteric lymph nodes and spleens upon *C. jejuni* infection of secondary abiotic Nod2^−/−^ mice

**Fig. 9:**
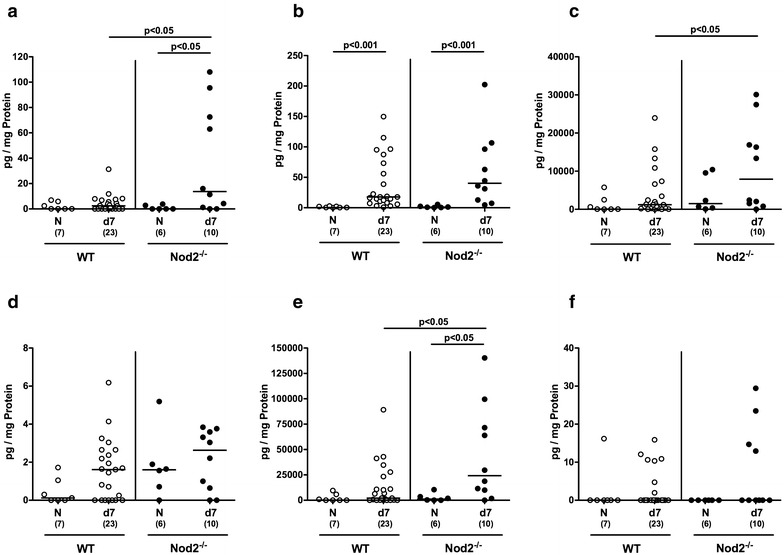
Secretion of pro- and anti-inflammatory cytokines in mesenteric lymph nodes of *C. jejuni* infected secondary abiotic Nod2^−/−^ mice. Secondary abiotic wildtype (WT; *white circles*) and Nod2^−/−^ mice (*black circles*) were generated by broad-spectrum antibiotic treatment and perorally infected with *C. jejuni* strain 81-176 by gavage at day (d) 0 and d1. **a** TNF, **b** IFN-γ, **c** MCP-1, **d** IL-12p70, **e** IL-6 and **f** IL-10 concentrations were determined in supernatants of ex vivo biopsies derived from mesenteric lymph nodes (MLN) at day 7 postinfection. Naive (N) secondary abiotic mice served as uninfected controls. Medians (*black bars*), level of significance (p-value) determined by Mann–Whitney U test and numbers of analyzed animals (in *parentheses*) are indicated. Data were pooled from three independent experiments

We next measured cytokine secretion in intestinal draining and systemic lymphatic tissues (i.e. MLN and spleen, respectively) following *C. jejuni* infection of secondary abiotic mice lacking Nod2. Whereas IFN-γ levels were elevated in MLN of both Nod2^−/−^ and WT mice at day 7 p.i. (p < 0.001; Fig. 9b), *C. jejuni* induced increases in TNF and IL-6 secretion could be observed in MLN of Nod2^−/−^ mice only (p < 0.05; Fig. 9a, e). At day 7 p.i., TNF, MCP-1 and IL-6 levels were higher in MLN of Nod2^−/−^ as compared to WT mice (p < 0.05; Fig. 9a, c, e). Notably, IL-10 secretion was virtually unaffected by *C. jejuni* infection (n.s.; Fig. 9f). Hence, distinct pro-inflammatory cytokine were higher in the intestines draining MLN of *C. jejuni* infected Nod2^−/−^ as compared to WT mice. In the systemic lymphatic compartment, however, virtually no *C. jejuni* induced increases in splenic cytokine secretion could be detected (Additional file 8: Figure S8). Notably, basal IL-6 levels were higher in spleens of secondary abiotic Nod2^−/−^ mice than WT controls (p < 0.05; Additional file 8: Figure S8E).

## Discussion

In order to prevent the host from invading pathogens including *C. jejuni* and to successfully combat infection, a concerted action of pattern recognition receptors, distinct innate and adaptive immune cells, and evolving signaling pathways are crucial [8–10]. In the present study we shed further light onto the role of the bacterial MDP sensor Nod2 in *C. jejuni* infection of secondary abiotic mice. Notably, Nod2 deficiency did not impact gastrointestinal colonization properties of *C. jejuni*, given that both secondary abiotic Nod2^−/−^ and WT mice harbored the pathogen with similar counts of up to 10^9^ CFU per g colonic luminal content. This might appear somewhat surprising, given that Nod2 deficiency has been shown to be associated with altered expression of antimicrobial peptides such as defensins leading to an insufficient clearance of the pathogen [20, 35]. Despite the high pathogenic burden within the intestines, mice were macroscopically uncompromised and did not display overt clinical signs of campylobacteriosis such as wasting or bloody diarrhea. This is well in line with our previous infection studies in secondary abiotic mice that were deficient in matrix metalloproteinase (MMP) -2 or -9 [24, 36], in cytokines belonging to the IL-23/IL-22/IL-18 axis [32] or in innate immune receptors including Toll-like receptor (TLR) -4 or -9 and respective WT counterparts [8, 32]. Nevertheless, in our present and previous studies [8, 24, 32, 36], distinct *C. jejuni* induced microscopic changes within the intestinal tract such as accelerated colonic epithelial apoptosis and pronounced influx of immune cells to the site of infection could be observed. Whereas Nod2 deficiency had no impact on *C. jejuni* induced colonic histopathological changes and apoptosis development, infected Nod2^−/−^ mice exhibited more distinct regenerative properties counteracting potential cell damage than WT controls. Furthermore, adaptive immune cell populations such as T and B lymphocytes were less distinctly abundant in the large intestines of *C. jejuni* infected Nod2^−/−^ as compared to WT mice, which is in line with our very recent investigations in conventionally colonized Nod2^−/−^ [37] and Nod2^−/−^ mice lacking IL-10 [38]. Our present colonic immune cell data are contrasted by the observed large intestinal secretion of pro-inflammatory mediators, given that *C. jejuni* induced increases in nitric oxide, TNF and IFN-γ protein concentrations were even more pronounced in Nod2^−/−^ mice which also held true for increased colonic TNF expression in *C. jejuni* infected conventionally colonized Nod2^−/−^ versus to WT mice as shown previously [37]. Overall, standard deviations in pro-inflammatory cytokine concentrations were relative high in *C. jejuni* infected mice of either genotype. It is therefore tempting to speculate that the extent of against *C. jejuni* directed immune responses might differ individually in both Nod2^−/−^ and WT mice.

We have recently highlighted the importance of the IL-23/IL-22/IL-18 axis in murine *C. jejuni* infection [25, 30–32]. In the present study we could demonstrate that respective cytokines were differentially expressed upon *C. jejuni* infection of secondary abiotic Nod2^−/−^ mice. As for large intestinal pro-inflammatory cytokines, *C. jejuni* induced up-regulation of colonic IL-22 mRNA was more pronounced in Nod2^−/−^ as compared to WT mice, whereas conversely, IL-18 was down-regulated in the former, and both IL-23p19 and IL-18 mRNA were lower in infected Nod2^−/−^ versus WT mice. IL-22 is a member of the IL-10 cytokine family and exerts dichotomous function which depends on the respective compartment and the surrounding cytokine milieu [31, 39, 40]. Whereas IL-22 exerts anti-inflammatory properties in the large intestinal tract [40], it has pro-inflammatory functions in the small intestines [29, 41, 42]. Given that IL-22 has been proven effective in antimicrobial host defence against *C. jejuni* [31, 32], more pronounced increases in IL-22 mRNA levels observed in *C. jejuni* infected Nod2^−/−^ mice might therefore point towards more distinct anti-pathogenic and anti-inflammatory counter-regulatory measures within the well-orchestrated host immune responses upon *C. jejuni* infection. Opposed to IL-22, IL-23p19, the master regulator of IL-22 expression [43], as well as IL-18 that is known to amplify IL-22 production during intestinal inflammation [41] were lower in *C. jejuni* infected Nod2^−/−^ as compared to WT mice. This might be due to a potential negative feed-back loop regulation between respective cytokines.

Even though the large intestinal tract constitutes the major predilection site of *C. jejuni* infection [8, 23, 44, 45], we here additionally assessed pro-inflammatory cytokine secretion in the small intestine. Remarkably, as opposed to the colon, ileal nitric oxide, TNF, IFN-γ and IL-6 concentrations were lower in *C. jejuni* infected Nod2^−/−^ as compared to WT mice. It is hence tempting to speculate that Nod2 might have dichotomous functions depending on the respective intestinal tissue, its immunological prerequisites, surrounding cytokines and other intestinal luminal factors.

To date, data are rather conflicting regarding the role of Nod2 in intestinal inflammation including *C. jejuni* infection. Depending on the applied in vivo model Nod2 might either prevent from or even enhance colitis development in *C. jejuni* infected mice. For instance, in IL-10^−/−^ mice that had been pre-treated with antibiotics for 1 week, Nod2 was shown to be essential for controlling campylobacteriosis, given that Nod2^−/−^ IL-10^−/−^ mice exhibited an exacerbation of *C. jejuni* induced large intestinal inflammation [46]. In another study by Jamontt and colleagues [47], however, Nod2 was shown to promote IL-10^−/−^ colitis, given that Nod2^−/−^ IL-10^−/−^ mice were protected from colitis development.

Also in non-infection induced intestinal inflammation models data regarding the impact of Nod in disease development are inconclusive so far. Following adaptive transfer of Nod2^−/−^ T lymphocytes, immunodeficient mice developed less severe chronic colitis, for instance, pointing towards a rather disease-promoting feature of Nod2 signaling [12]. Conversely, MDP application could sufficiently prevent from 2,4,6-trinitro-benzene-sulfonic acid (TNBS) induced colitis, whereas MDP-mediated prevention of diseases was overruled in Nod2^−/−^ mice indicative for a protective role of Nod2 signaling [48]. In an own study we further demonstrated that Nod2 is involved in protection of mice from *Toxoplasma gondii* induced acute ileitis [49].

Epithelial barrier integrity is pivotal for preventing pathogenic translocation from the intestinal tract to extra-intestinal including systemic tissue sites with potentially fatal consequences [50]. Even though the mucin MUC2 was down-regulated in *C. jejuni* infected Nod2^−/−^ mice, this did not result in increased pathogenic translocation rates to liver, kidney or spleen, whereas viable *C. jejuni* could be isolated in comparable numbers from MLN of all infected Nod2^−/−^ and WT mice. Interestingly, secretion of pro-inflammatory cytokine including TNF, MCP-1 and IL-6 were higher in MLN of *C. jejuni* infected Nod2^−/−^ as compared to WT mice. In the systemic lymphatic compartment, however, virtually no *C. jejuni* induced increases in cytokine secretion could be detected.

*In conclusion*, upon *C. jejuni* infection of secondary abiotic mice Nod2 signaling is involved in the initiation of a well-orchestrated innate and adaptive immune response but does not limit pathogen colonization. Future studies need to dissect the exact regulatory interactions to improve our understanding of the molecular mechanisms underlying campylobacteriosis.

## Additional files

**Additional file 1: Figure S1.** Fecal blood in *C. jejuni* infected secondary abiotic Nod2^−/−^ mice. Secondary abiotic wildtype (WT; white circles) and Nod2^−/−^ mice (black circles) were generated by broad-spectrum antibiotic treatment and perorally infected with *C. jejuni* strain 81-176 by gavage at day (d) 0 and d1. Microscopic or macroscopic occurrence of blood in fecal samples before and after infection was assessed applying a standardized haemoccult scoring system (see methods). Absolute numbers of animals with blood-positive fecal samples out of the total number of analyzed mice are indicated (in parentheses). Data were pooled from four independent experiments.

**Additional file 2: Figure S2.** Colonic histopathological changes in *C. jejuni* infected secondary abiotic Nod2^−/−^ mice. Secondary abiotic wildtype (WT, upper panel) and Nod2^−/−^ mice (lower panel) were generated by broad-spectrum antibiotic treatment and perorally infected with *C. jejuni* strain 81-176 by gavage at day (d) 0 and d1. Photomicrographs representative for four independent experiments (x100 magnification, scale bar 100 μm) depict histopathological mucosal changes in H&E stained large intestinal paraffin sections at day 7 following *C. jejuni* infection (d7, right panel). Naive secondary abiotic mice (left panel) served as uninfected controls.

**Additional file 3: Figure S3.** Colonic epithelial apoptosis in *C. jejuni* infected secondary abiotic Nod2^−/−^ mice. Secondary abiotic wildtype (WT, upper panel) and Nod2^−/−^ mice (lower panel) were generated by broad-spectrum antibiotic treatment and perorally infected with *C. jejuni* strain 81-176 by gavage at day (d) 0 and d1. Photomicrographs representative for four independent experiments (x400 magnification, scale bar 20 μm) depict apoptotic (caspase3 positive) cells (arrows) in large intestinal epithelia at day 7 following *C. jejuni* infection (d7, right panel) applying *in situ* immunohistochemistry of colonic paraffin sections. Naive secondary abiotic mice (left panel) served as uninfected controls.

**Additional file 4: Figure S4.** Proliferating colonic epithelial cells in *C. jejuni* infected secondary abiotic Nod2^−/−^ mice. Secondary abiotic wildtype (WT, upper panel) and Nod2^−/−^ mice (lower panel) were generated by broad-spectrum antibiotic treatment and perorally infected with *C. jejuni* strain 81-176 by gavage at day (d) 0 and d1. Photomicrographs representative for four independent experiments (x100 magnification, scale bar 100 μm) depict apoptotic (Ki67 positive) cells in large intestinal epithelia at day 7 following *C. jejuni* infection (d7, right panel) applying *in situ* immunohistochemistry of colonic paraffin sections. Naive secondary abiotic mice (left panel) served as uninfected controls.

**Additional file 5: Figure S5.** Colonic T lymphocytes in *C. jejuni* infected secondary abiotic Nod2^−/−^ mice. Secondary abiotic wildtype (WT, upper panel) and Nod2^−/−^ mice (lower panel) were generated by broad-spectrum antibiotic treatment and perorally infected with *C. jejuni* strain 81-176 by gavage at day (d) 0 and d1. Photomicrographs representative for four independent experiments (x400 magnification, scale bar 20 μm) depict colonic CD3 positive T lymphocytes at day 7 following *C. jejuni* infection (d7, right panel) applying *in situ* immunohistochemistry of colonic paraffin sections. Naive secondary abiotic mice (left panel) served as uninfected controls.

**Additional file 6: Figure S6.** Colonic regulatory T cells in *C. jejuni* infected secondary abiotic Nod2^−/−^ mice. Secondary abiotic wildtype (WT, upper panel) and Nod2^−/−^ mice (lower panel) were generated by broad-spectrum antibiotic treatment and perorally infected with *C. jejuni* strain 81-176 by gavage at day (d) 0 and d1. Photomicrographs representative for four independent experiments (x400 magnification, scale bar 20 μm) depict colonic FOXP3 positive regulatory T cells at day 7 following *C. jejuni* infection (d7, right panel) applying *in situ* immunohistochemistry of colonic paraffin sections. Naive secondary abiotic mice (left panel) served as uninfected controls.

**Additional file 7: Figure S7.** Colonic B lymphocytes in *C. jejuni* infected secondary abiotic Nod2^−/−^ mice. Secondary abiotic wildtype (WT, upper panel) and Nod2^−/−^ mice (lower panel) were generated by broad-spectrum antibiotic treatment and perorally infected with *C. jejuni* strain 81-176 by gavage at day (d) 0 and d1. Photomicrographs representative for four independent experiments (x400 magnification, scale bar 20 μm) depict colonic B220 positive B lymphocytes at day 7 following *C. jejuni* infection (d7, right panel) applying *in situ* immunohistochemistry of colonic paraffin sections. Naive secondary abiotic mice (left panel) served as uninfected controls.

**Additional file 8: Figure S8.** Secretion of pro- and anti-inflammatory cytokines in spleens of *C. jejuni* infected secondary abiotic Nod2^−/−^ mice. Secondary abiotic wildtype (WT; white circles) and Nod2^−/−-^ mice (black circles) were generated by broad-spectrum antibiotic treatment and perorally infected with *C. jejuni* strain 81-176 by gavage at day (d) 0 and d1. **(A)** Nitric oxide, **(B)** TNF, **(C)** IFN-γ, **(D)** MCP-1, **(E)** IL-6 and **(F)** IL-10 concentrations were determined in supernatants of *ex vivo* biopsies derived from spleens at day 7 postinfection. Naive (N) secondary abiotic mice served as uninfected controls. Medians (black bars), level of significance (p-value) determined by Mann-Whitney U test and numbers of analyzed animals (in parentheses) are indicated. Data were pooled from four independent experiments.

## Abbreviations

CFU: colony forming units
HPF: high power field
HPRT: hypoxanthine-phosphoribosyltransferase
MDP: muramyl dipeptide
MLN: mesenteric lymph nodes
MMP: matrix metalloproteinase
MUC2: mucin-2
NOD: nucleotide-binding oligomerization domain
PCR: polymerase chain reaction
PBS: phosphate buffered saline
p.i.: postinfection
SPF: special pathogen-free
TLR: Toll-like receptor
TNBS: trinitrobenzene sulfonic acid
Treg: regulatory T cells
WT: wildtype
