# Supplementary figures and images for: Immune responses upon Campylobacter jejuni infection of secondary abiotic mice lacking nucleotide-oligomerization-domain-2
Source: Gut Pathog. 2017 Jun 6;9:33. doi: 10.1186/s13099-017-0182-0 (PMC5461728; doi:10.1186/s13099-017-0182-0)

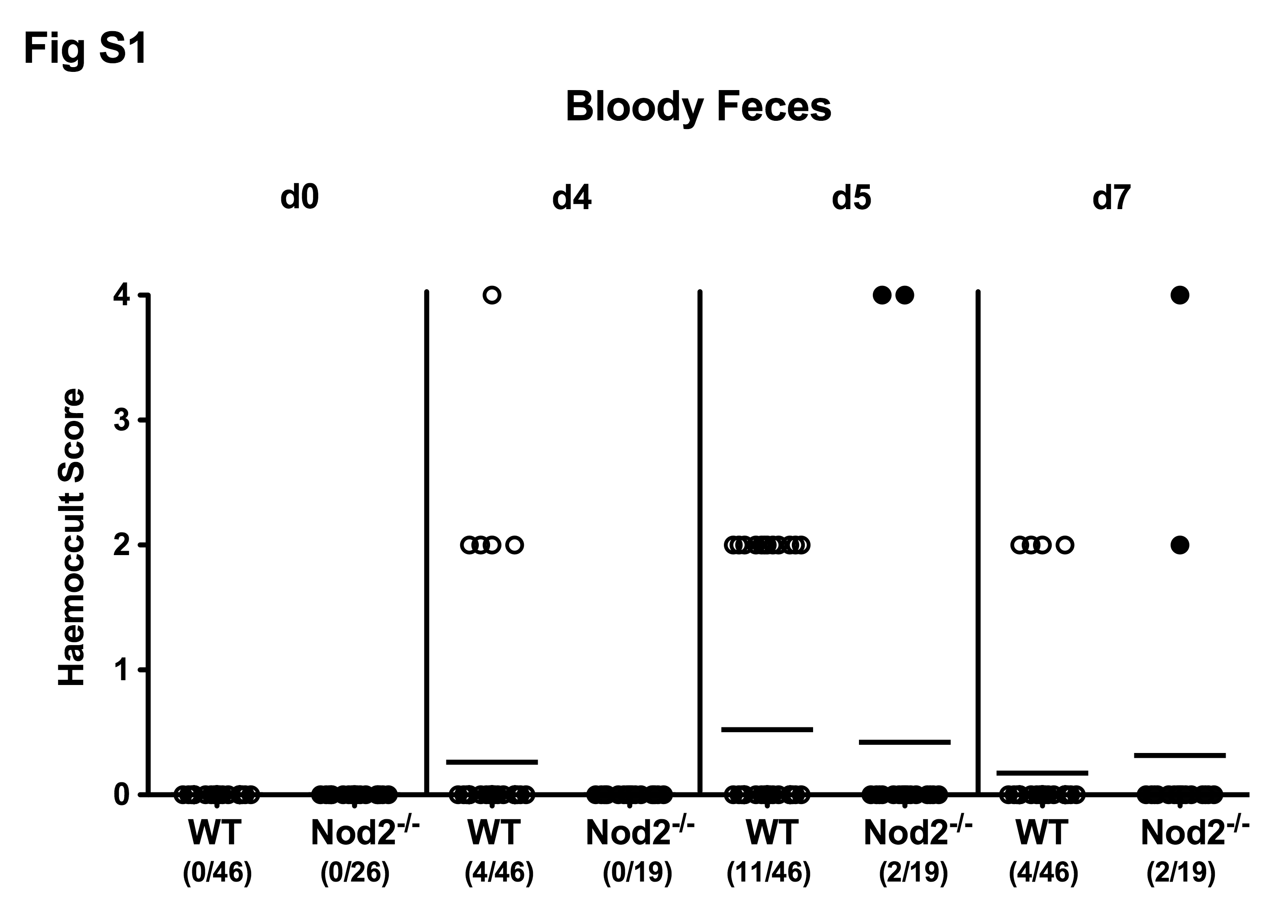

Supplement: Supplementary file 1 — Additional file 1: Figure S1. Fecal blood in C. jejuni infected secondary abiotic Nod2−/− mice. Secondary abiotic wildtype (WT; white circles) and Nod2−/− mice (black circles) were generated by broad-spectrum antibiotic treatment and perorally infected with C. jejuni strain 81-176 by gavage at day (d) 0 and d1. Microscopic or macroscopic occurrence of blood in fecal samples before and after infection was assessed applying a standardized haemoccult scoring system (see methods). Absolute numbers of animals with blood-positive fecal samples out of the total number of analyzed mice are indicated (in parentheses). Data were pooled from four independent experiments. [file 13099_2017_182_MOESM1_ESM.tiff]

**Fig S2**

**Naive**

**d7**

**WT**

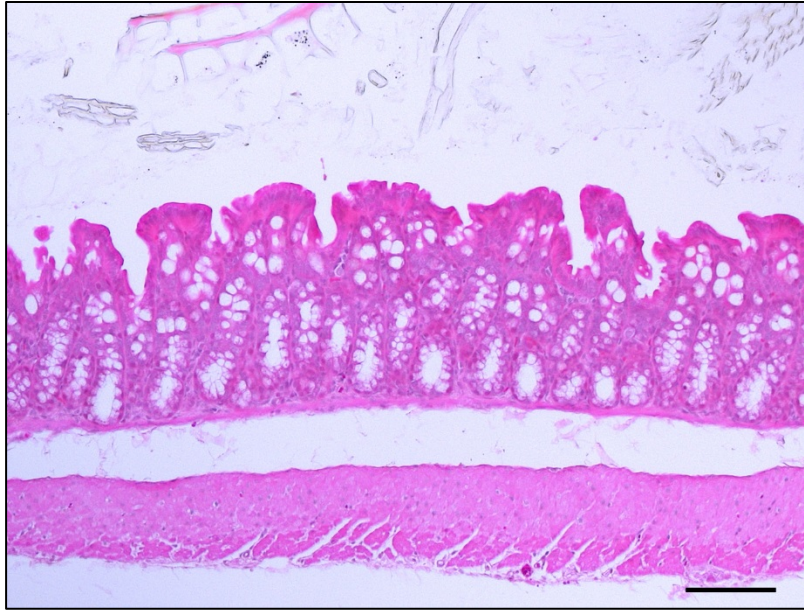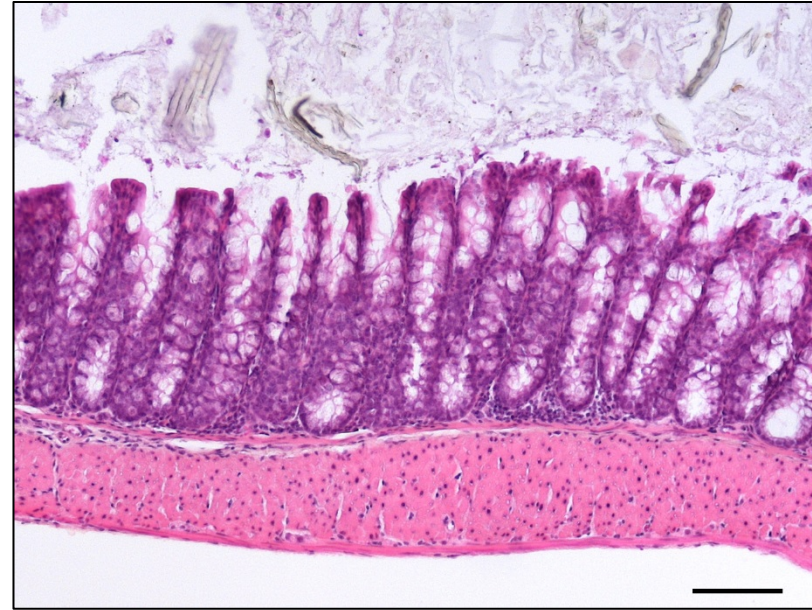

**Nod2<sup>-/-</sup>**

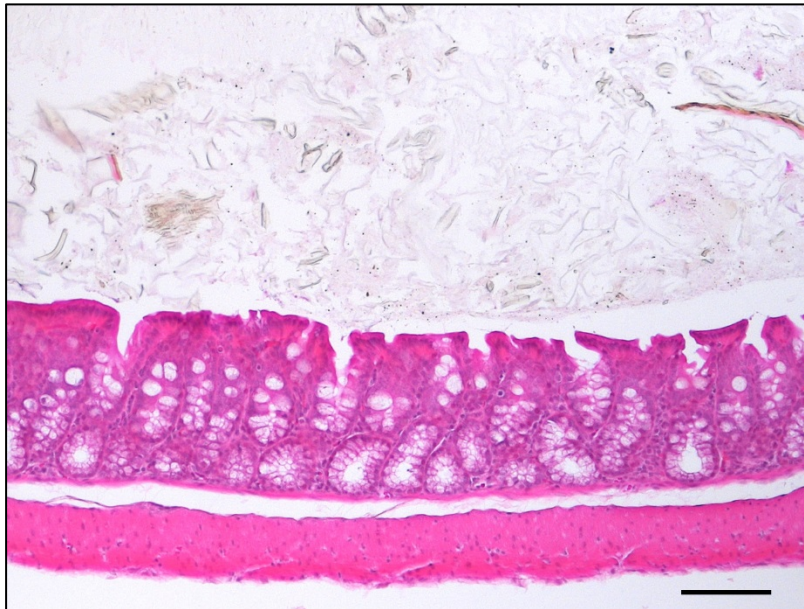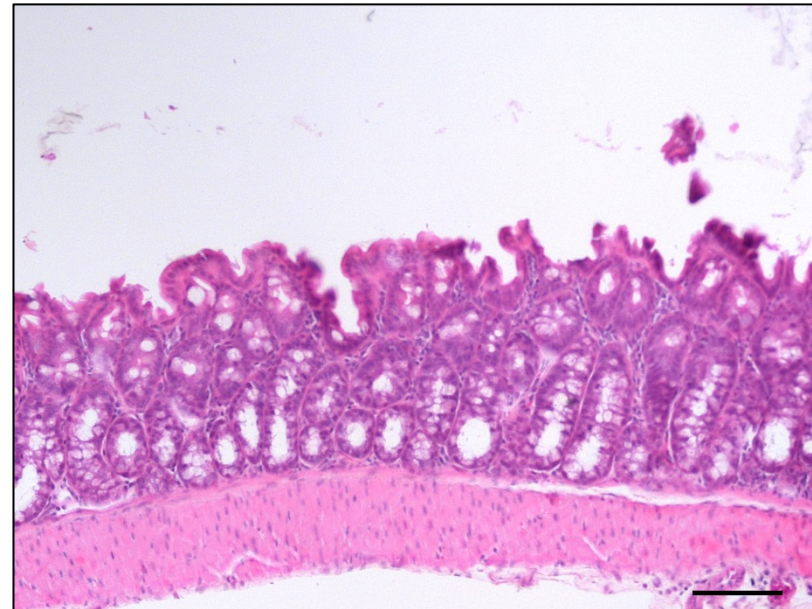

Supplement: Supplementary file 2 — Additional file 2: Figure S2. Colonic histopathological changes in C. jejuni infected secondary abiotic Nod2−/− mice. Secondary abiotic wildtype (WT, upper panel) and Nod2−/− mice (lower panel) were generated by broad-spectrum antibiotic treatment and perorally infected with C. jejuni strain 81-176 by gavage at day (d) 0 and d1. Photomicrographs representative for four independent experiments (x100 magnification, scale bar 100 μm) depict histopathological mucosal changes in H&E stained large intestinal paraffin sections at day 7 following C. jejuni infection (d7, right panel). Naive secondary abiotic mice (left panel) served as uninfected controls. [file 13099_2017_182_MOESM2_ESM.pdf]

**Fig S3**

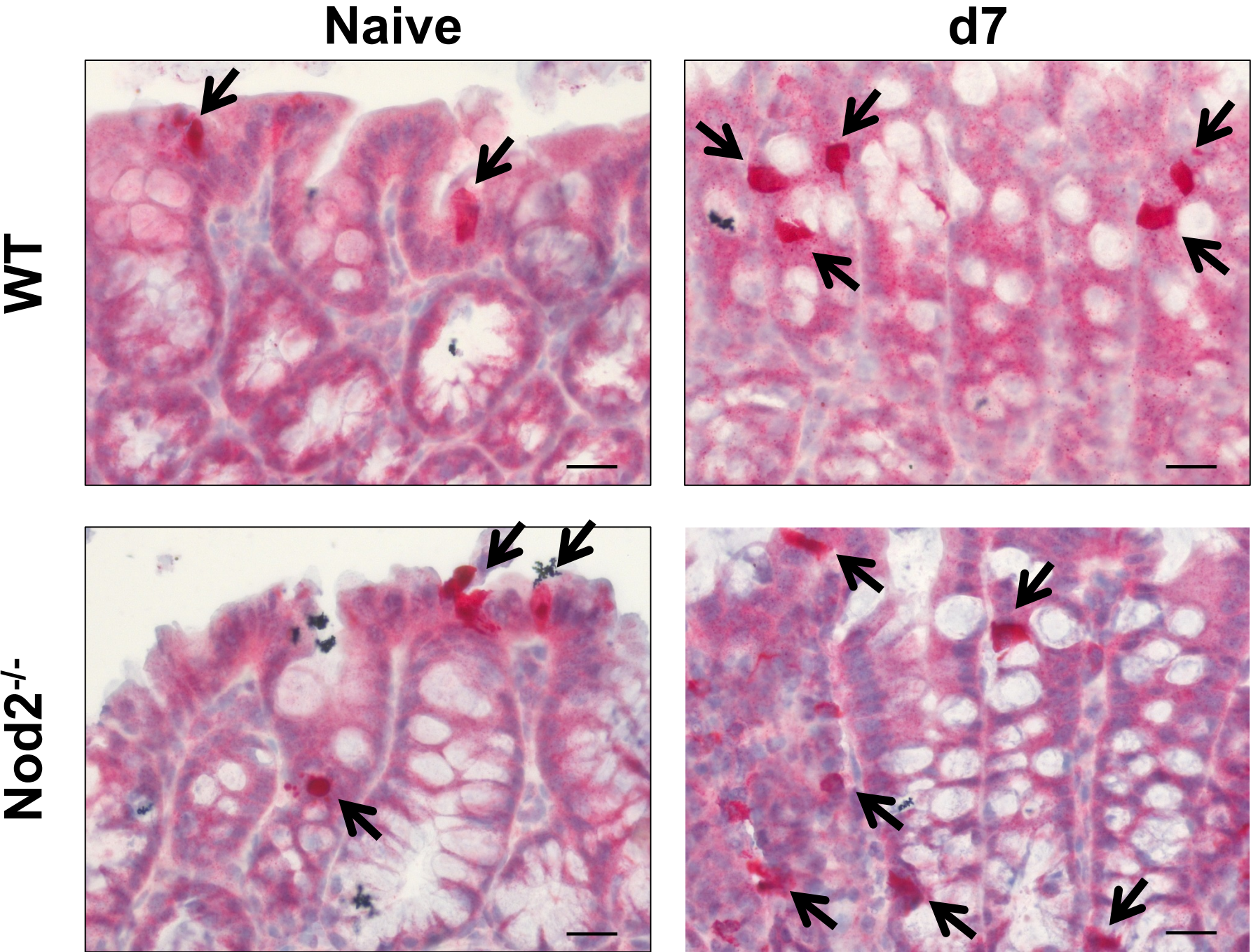

Supplement: Supplementary file 3 — Additional file 3: Figure S3. Colonic epithelial apoptosis in C. jejuni infected secondary abiotic Nod2−/− mice. Secondary abiotic wildtype (WT, upper panel) and Nod2−/− mice (lower panel) were generated by broad-spectrum antibiotic treatment and perorally infected with C. jejuni strain 81-176 by gavage at day (d) 0 and d1. Photomicrographs representative for four independent experiments (x400 magnification, scale bar 20 μm) depict apoptotic (caspase3 positive) cells (arrows) in large intestinal epithelia at day 7 following C. jejuni infection (d7, right panel) applying in situ immunohistochemistry of colonic paraffin sections. Naive secondary abiotic mice (left panel) served as uninfected controls. [file 13099_2017_182_MOESM3_ESM.pdf]

**Fig S4**

**Naive**

**d7**

**WT**

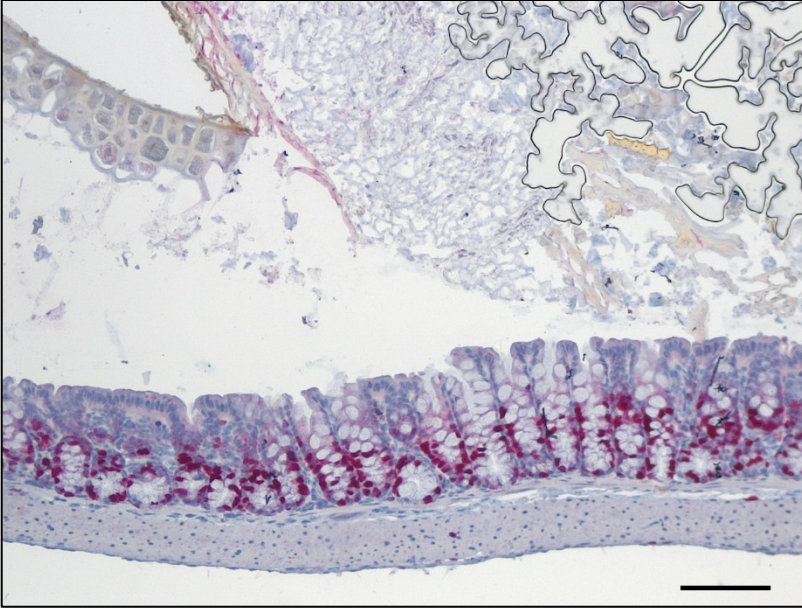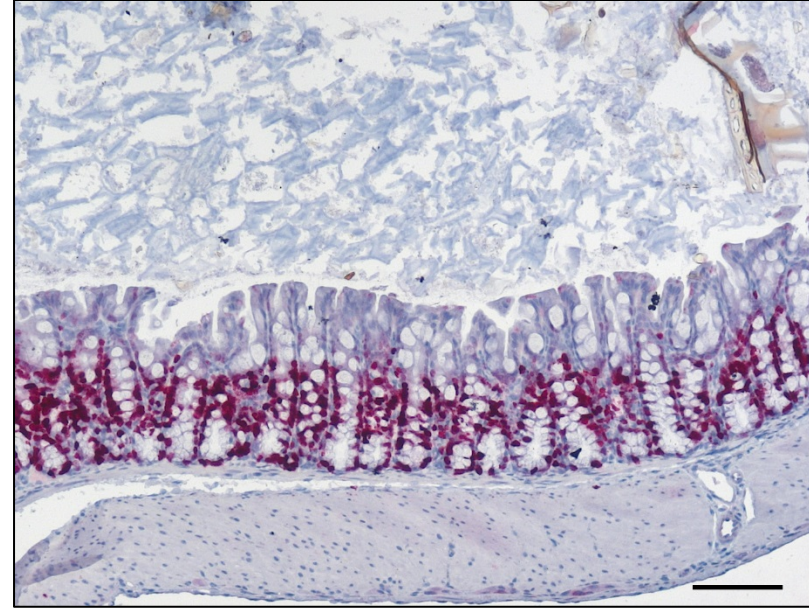

**Nod2<sup>-/-</sup>**

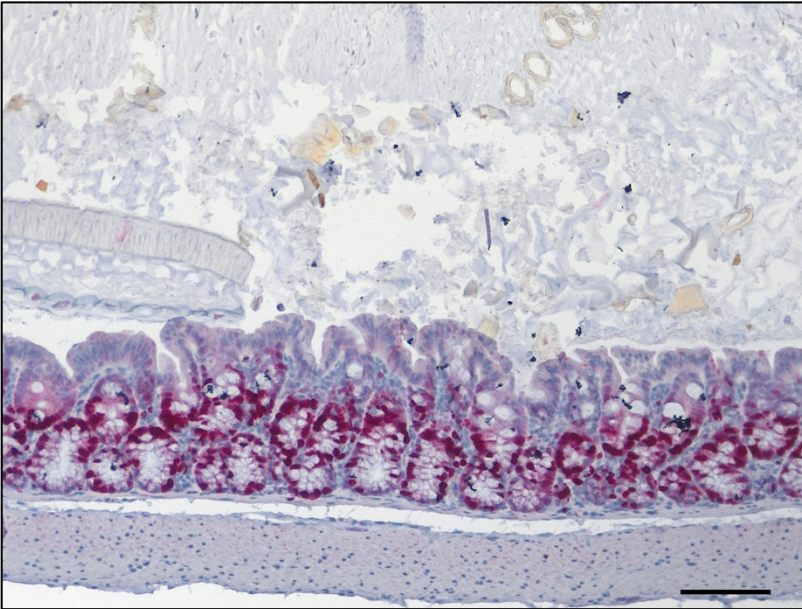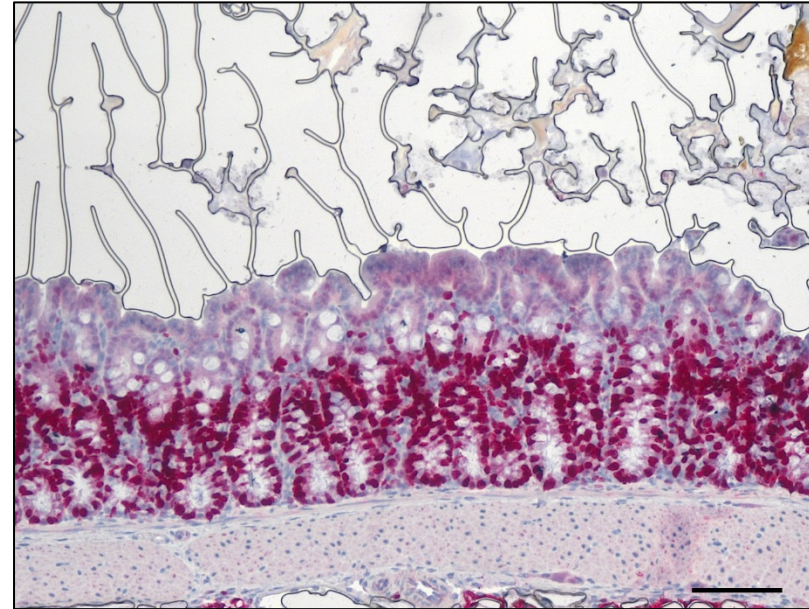

Supplement: Supplementary file 4 — Additional file 4: Figure S4. Proliferating colonic epithelial cells in C. jejuni infected secondary abiotic Nod2−/− mice. Secondary abiotic wildtype (WT, upper panel) and Nod2−/− mice (lower panel) were generated by broad-spectrum antibiotic treatment and perorally infected with C. jejuni strain 81-176 by gavage at day (d) 0 and d1. Photomicrographs representative for four independent experiments (x100 magnification, scale bar 100 μm) depict apoptotic (Ki67 positive) cells in large intestinal epithelia at day 7 following C. jejuni infection (d7, right panel) applying in situ immunohistochemistry of colonic paraffin sections. Naive secondary abiotic mice (left panel) served as uninfected controls. [file 13099_2017_182_MOESM4_ESM.pdf]

**Fig S5**

**Naive**

**d7**

**WT**

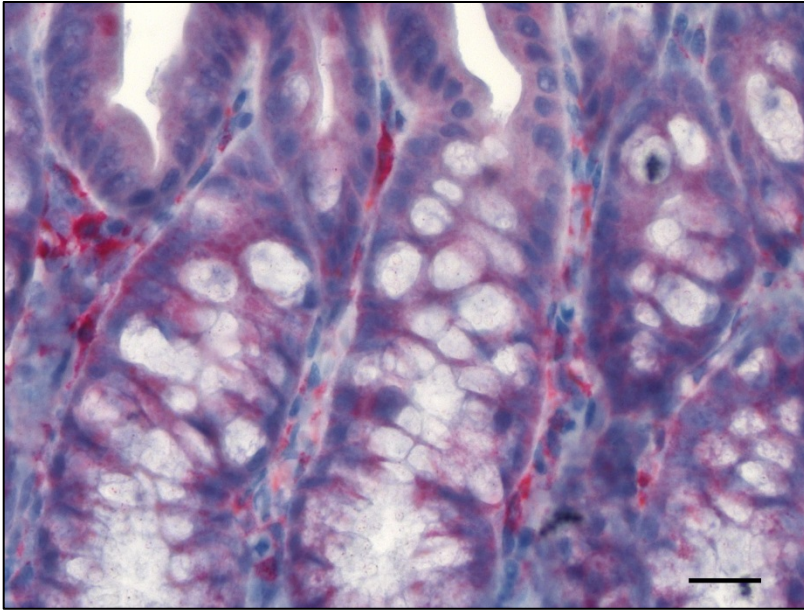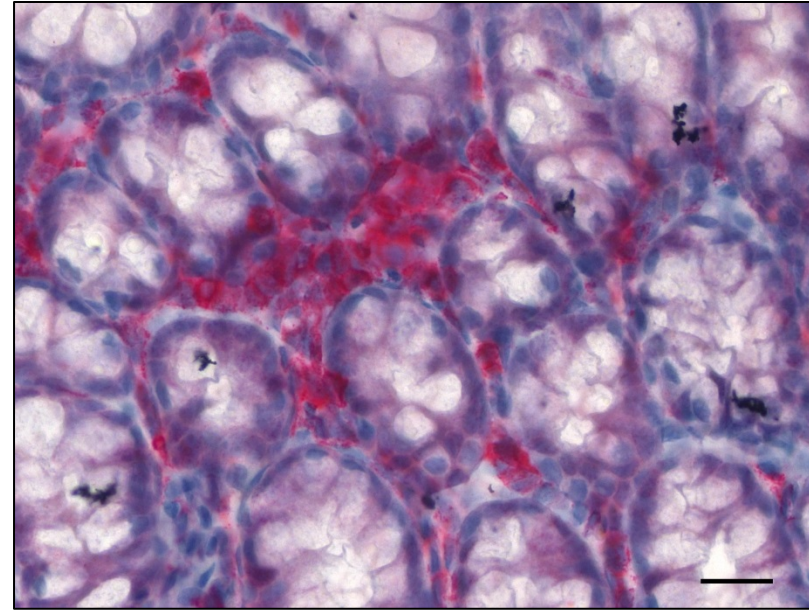

**Nod2<sup>-/-</sup>**

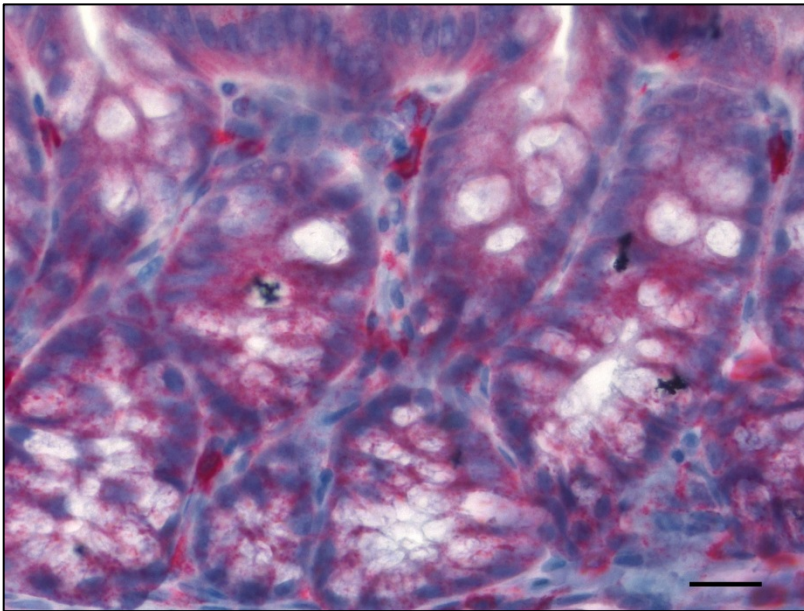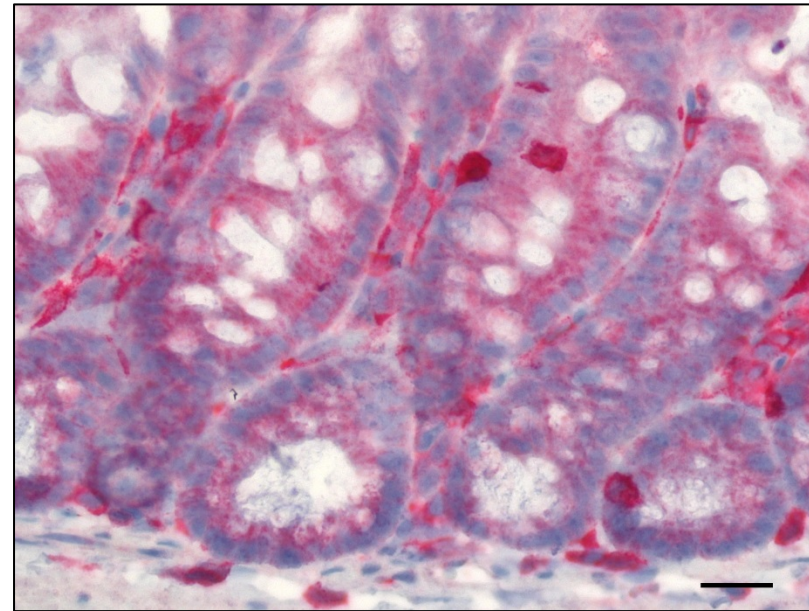

Supplement: Supplementary file 5 — Additional file 5: Figure S5. Colonic T lymphocytes in C. jejuni infected secondary abiotic Nod2−/− mice. Secondary abiotic wildtype (WT, upper panel) and Nod2−/− mice (lower panel) were generated by broad-spectrum antibiotic treatment and perorally infected with C. jejuni strain 81-176 by gavage at day (d) 0 and d1. Photomicrographs representative for four independent experiments (x400 magnification, scale bar 20 μm) depict colonic CD3 positive T lymphocytes at day 7 following C. jejuni infection (d7, right panel) applying in situ immunohistochemistry of colonic paraffin sections. Naive secondary abiotic mice (left panel) served as uninfected controls. [file 13099_2017_182_MOESM5_ESM.pdf]

**Fig S6**

**Naive**

**d7**

**WT**

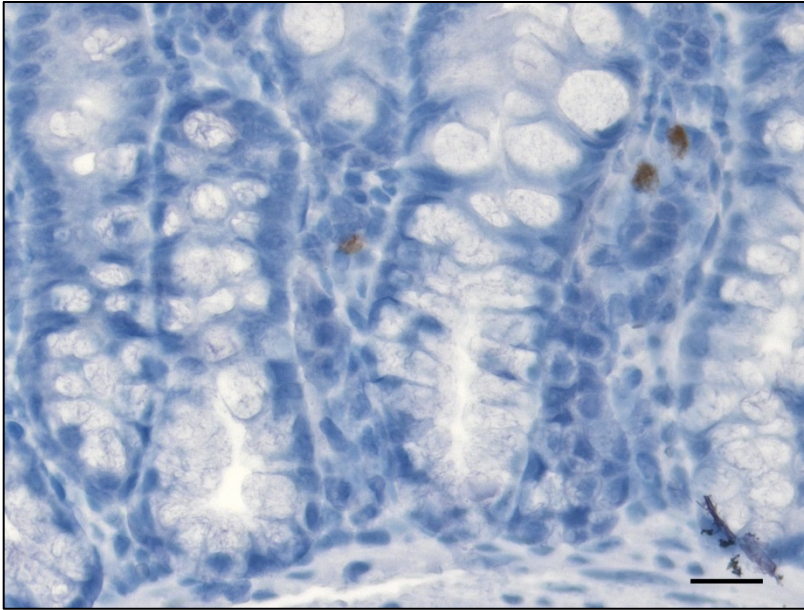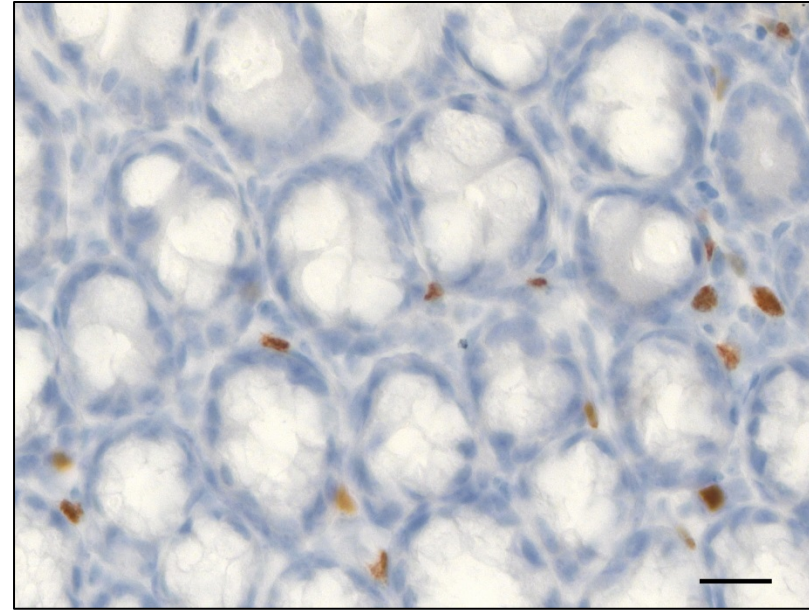

**Nod2<sup>-/-</sup>**

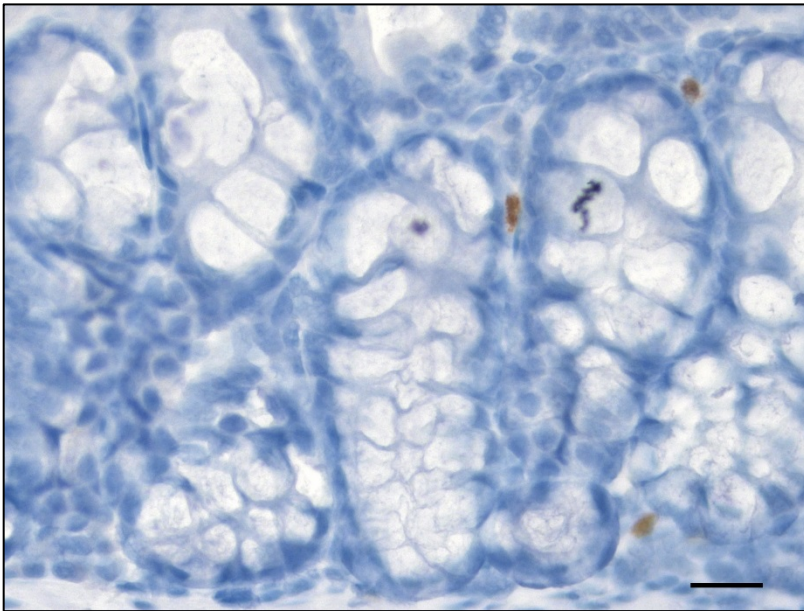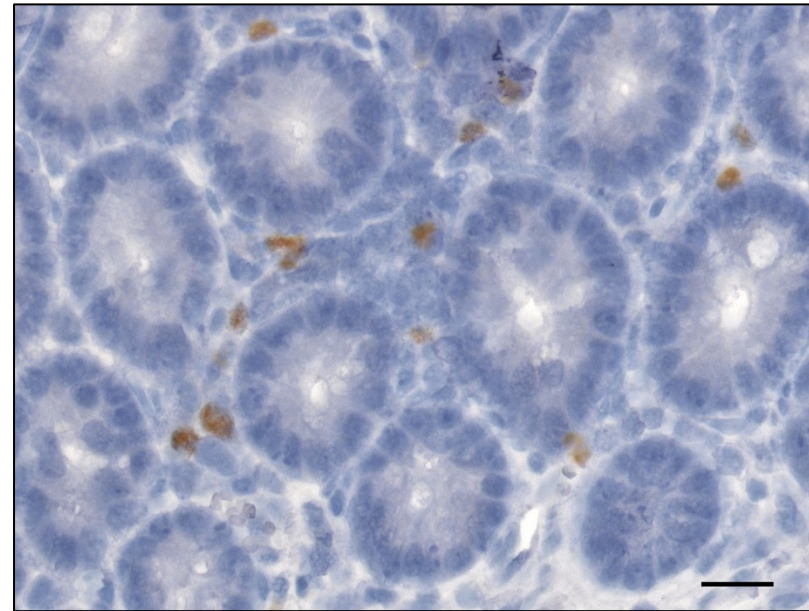

Supplement: Supplementary file 6 — Additional file 6: Figure S6. Colonic regulatory T cells in C. jejuni infected secondary abiotic Nod2−/− mice. Secondary abiotic wildtype (WT, upper panel) and Nod2−/− mice (lower panel) were generated by broad-spectrum antibiotic treatment and perorally infected with C. jejuni strain 81-176 by gavage at day (d) 0 and d1. Photomicrographs representative for four independent experiments (x400 magnification, scale bar 20 μm) depict colonic FOXP3 positive regulatory T cells at day 7 following C. jejuni infection (d7, right panel) applying in situ immunohistochemistry of colonic paraffin sections. Naive secondary abiotic mice (left panel) served as uninfected controls. [file 13099_2017_182_MOESM6_ESM.pdf]

**Fig S7**

**Naive**

**d7**

**WT**

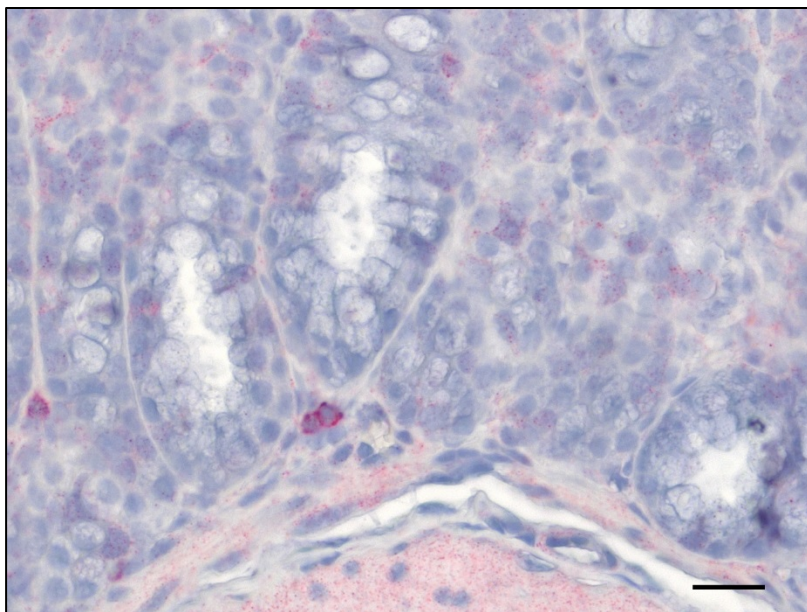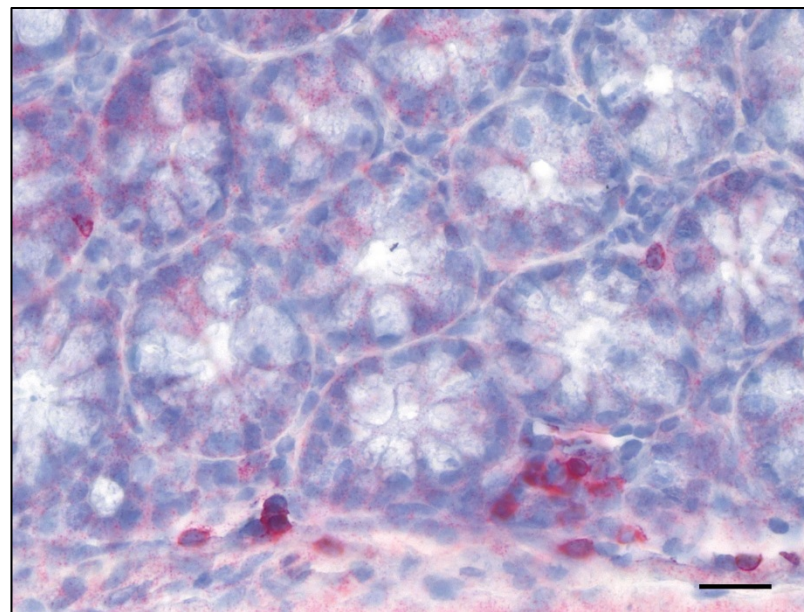

**Nod2<sup>-/-</sup>**

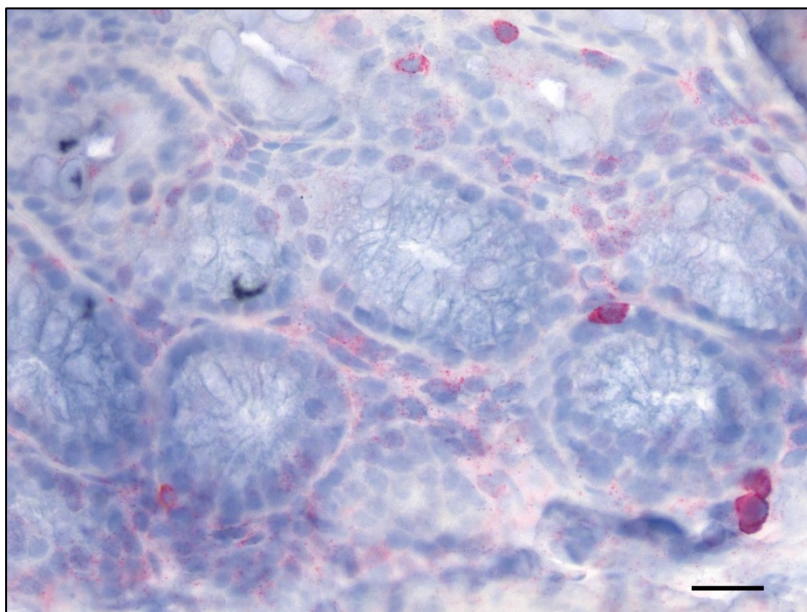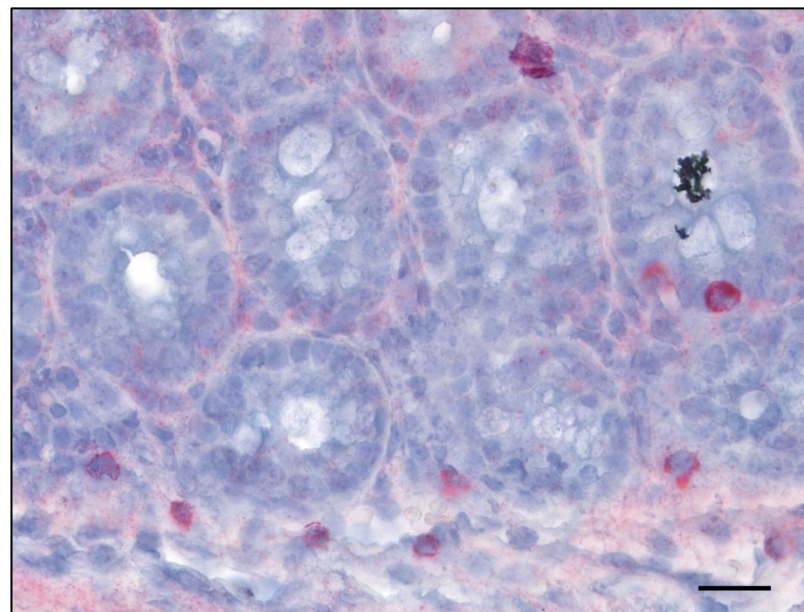

Supplement: Supplementary file 7 — Additional file 7: Figure S7. Colonic B lymphocytes in C. jejuni infected secondary abiotic Nod2−/− mice. Secondary abiotic wildtype (WT, upper panel) and Nod2−/− mice (lower panel) were generated by broad-spectrum antibiotic treatment and perorally infected with C. jejuni strain 81-176 by gavage at day (d) 0 and d1. Photomicrographs representative for four independent experiments (x400 magnification, scale bar 20 μm) depict colonic B220 positive B lymphocytes at day 7 following C. jejuni infection (d7, right panel) applying in situ immunohistochemistry of colonic paraffin sections. Naive secondary abiotic mice (left panel) served as uninfected controls. [file 13099_2017_182_MOESM7_ESM.pdf]

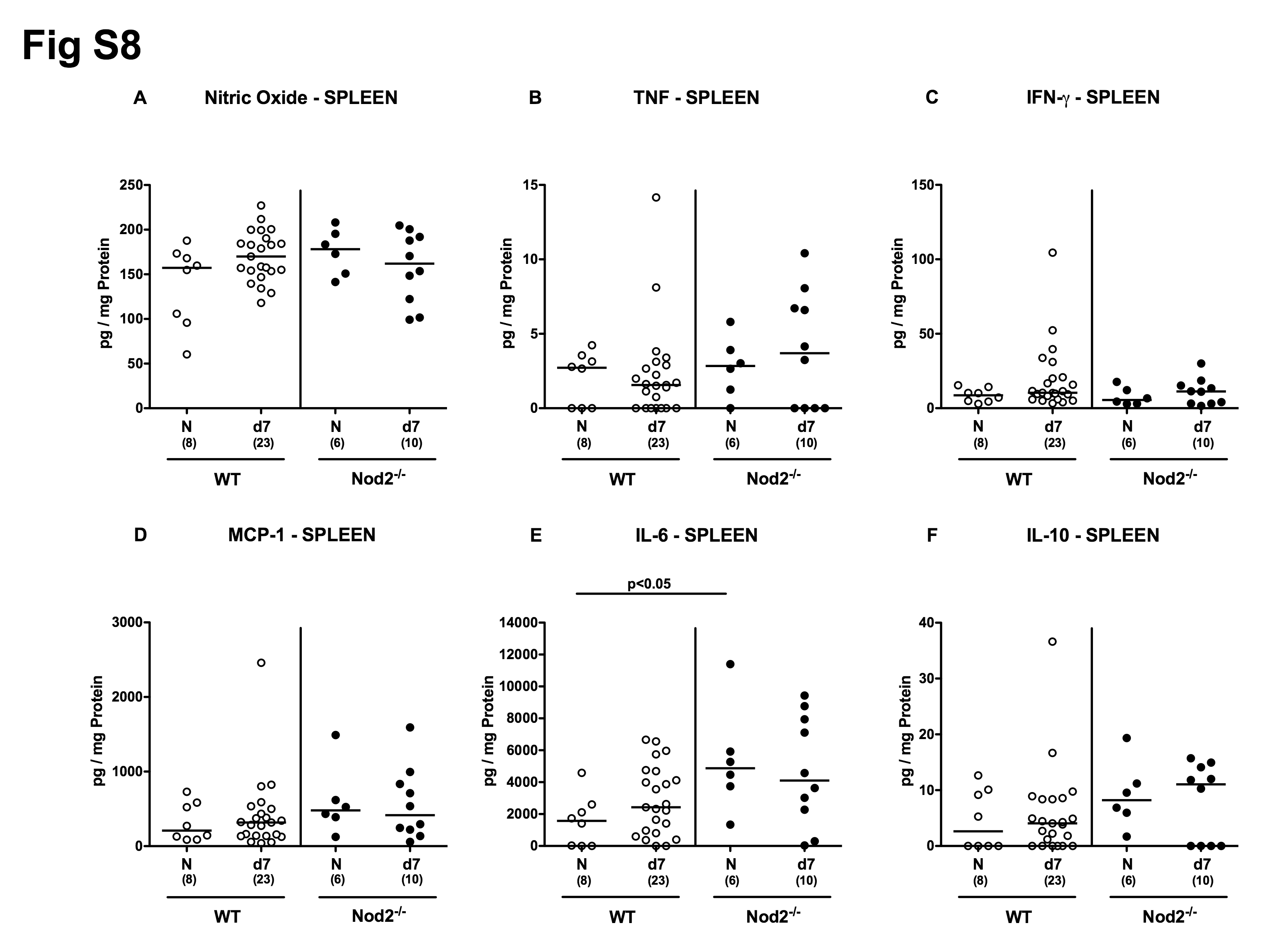

Supplement: Supplementary file 8 — Additional file 8: Figure S8. Secretion of pro- and anti-inflammatory cytokines in spleens of C. jejuni infected secondary abiotic Nod2−/− mice. Secondary abiotic wildtype (WT; white circles) and Nod2−/−- mice (black circles) were generated by broad-spectrum antibiotic treatment and perorally infected with C. jejuni strain 81-176 by gavage at day (d) 0 and d1. (A) Nitric oxide, (B) TNF, (C) IFN-γ, (D) MCP-1, (E) IL-6 and (F) IL-10 concentrations were determined in supernatants of ex vivo biopsies derived from spleens at day 7 postinfection. Naive (N) secondary abiotic mice served as uninfected controls. Medians (black bars), level of significance (p-value) determined by Mann-Whitney U test and numbers of analyzed animals (in parentheses) are indicated. Data were pooled from four independent experiments. [file 13099_2017_182_MOESM8_ESM.tiff]
